# Seeking genetic signature of radiosensitivity - a novel method for data analysis in case of small sample sizes

**DOI:** 10.1186/1742-4682-11-S1-S2

**Published:** 2014-05-07

**Authors:** Joanna Zyla, Paul Finnon, Robert Bulman, Simon Bouffler, Christophe Badie, Joanna Polanska

**Affiliations:** 1 Data Mining Group, Faculty of Automatic Control, Electronic and Computer Science, Silesian University of Technology, Akademicka 16, 44-100 Gliwice, Poland; 2 Public Health England, OX11 ORQ Chilton, Didcot, UK

**Keywords:** radiosensitivity, polymorphisms, chromosomal abberations, data mining, mathematical modelling, GWAS

## Abstract

**Background:**

The identification of polymorphisms and/or genes responsible for an organism's radiosensitivity increases the knowledge about the cell cycle and the mechanism of the phenomena themselves, possibly providing the researchers with a better understanding of the process of carcinogenesis.

**Aim:**

The aim of the study was to develop a data analysis strategy capable of discovering the genetic background of radiosensitivity in the case of small sample size studies.

**Results:**

Among many indirect measures of radiosensitivity known, the level of radiation-induced chromosomal aberrations was used in the study. Mathematical modelling allowed the transformation of the yield-time curve of radiation-induced chromosomal aberrations into the exponential curve with limited number of parameters, while Gaussian mixture models applied to the distributions of these parameters provided the criteria for mouse strain classification. A detailed comparative analysis of genotypes between the obtained subpopulations of mice followed by functional validation provided a set of candidate polymorphisms that might be related to radiosensitivity. Among 1857 candidate relevant SNPs, that cluster in 28 genes, eight SNPs were detected nonsynonymous (nsSNP) on protein function. Two of them, rs48840878 (gene Msh3) and rs5144199 (gene Cc2d2a), were predicted as having increased probability of a deleterious effect. Additionally, rs48840878 is capable of disordering phosphorylation with 14 PKs. In silico analysis of candidate relevant SNP similarity score distribution among 60 CGD mouse strains allowed for the identification of SEA/GnJ and ZALENDE/EiJ mouse strains (95.26% and 86.53% genetic consistency respectively) as the most similar to radiosensitive subpopulation

**Conclusions:**

A complete step-by-step strategy for seeking the genetic signature of radiosensitivity in the case of small sample size studies conducted on mouse models was proposed. It is shown that the strategy, which is a combination of mathematical modelling, statistical analysis and data mining methodology, allows for the discovery of candidate polymorphisms which might be responsible for radiosensitivity phenomena.

## Introduction

Radiosensitivity is the relative susceptibility of cells, tissues, organs or organisms to the harmful effect of radiation. Effects of radiation include, among the others, DNA mutations, of which those observed in genes responsible for DNA repair (e.g. XRCC1 in base excision repair) are believed to be the most dangerous ones from a radiosensitivity point of view [1]. Nowadays, low-dose radiation and its effect (not immediately noticeable) is a leading topic because of the problems with collecting reliable data and its long-term biological consequence [2]. As with sensitivity to sunlight or to chemotherapeutic drugs, sensitivity to ionizing radiation shows variation among individuals [3]. It has been shown that radiosensitivity is a heritable trait of our organisms shaped by environmental factors [4]. The quantification of the cancer risk associated with ionising radiation requires mapping and identification of the genes that affect risk. This will eventually lead to the prediction of individual sensitivity. Although a large amount of data has already been obtained, the identification of genes potentially involved in radiosensitivity for the prediction of individual cancer risk is not complete and further analyses are required. The majority of studies reported in the literature are of the Genome-Wide Association Studies (GWAS) type, where the inter-individual variability must be considered in a process of data analysis. By definition, GWAS projects require both, a large number of polymorphisms, usually Single Nucleotide Polymorphisms (SNPs), and a large number of individuals to be analysed to achieve the required level of Family-Wise-Error-Rate (FWER) [5,6]. An alternative strategy is that the a priori chosen candidate SNPs are analysed in a smaller group of individuals, which allows the following of some signal pathways in the organisms in detail [7]. The experimental design used in our study is a hybrid of these two. The number of SNPs analysed stays large (even higher than in regular GWAS projects), but the sample consists of the animals selected from the inbred mouse strains, which results in the limitation of the overall inter-individual variability.

## Materials and methods

### Materials

The sample consisted of 14 inbred mouse strains. The number of biological replicates per mouse strain ranged from 1 to 3 (Table 1). All investigated mice were females approximately 10-12 weeks old when they were euthanised. The sensitivity to irradiation was indirectly assessed with the use of the G2 chromosomal radiosensitivity assay (G2CR) [8-11]. Enhanced G(2)chromosomal radiosensitivity is a consequence of inherited defects in the ability of cells to process DNA damage from endogenous or exogenous sources, of a type that is mimicked by ionizing radiation, and that such defects predispose to breast and colorectal cancer [12-16]. Lipopolysaccharide/Concanavalin A were isolated and grew up in RPMI for 48h. Proliferating cells in G2 phase were extracted and ex-vivo irradiated with a dose of 1.5 Gy. The irradiations were performed using a Siemans Stabiliplan-1 X-ray set, running at 250V (constant potential) with a Cu/Al filter producing a beam of 1.2 mm HVL Cu at a dose rate of 0.73 Gyˆ-1. The number of chromatid breaks and gaps was counted and normalized per 100 cells from one mouse. Colcemide was added at 1, 2, 3, 4 and 5 hours post-irradiation with cells harvested 1 hour later to collect the measurements. The relative number of chromosomal aberrations, defined as the difference between after and before irradiation G2CR values was used for further analyses [17]. The data on genome-wide single nucleotide polymorphisms (SNPs) were collected from the Center for Genome Dynamics Mouse SNP Database (CGD SNP database) [18] and consisted of the information on 7,849,649 polymorphisms genotyped for all analysed mouse strains (Table 2).

**Table 1:** List of mouse strains tested in G2CR assay with a number of biological replicates.

Mouse Strain	No. of biological replication	Mouse Strain	No. of biological replication
A/J	3	C57Bl/6J	2
AKR/J	1	DBA/2J	1
Balb/cAn	1	LP	2
Balb/cByJ	3	NOD/LtJ	1
C3H/HeHsd	1	NON/LtJ	2
CBA/Ca	1	NZB/B1NJ	1
CBA/H	3	SJL/J	1

**Table 2:** Number of SNPs (loci) genotyped for all analysed mouse strains.

Chromosome	No. of SNPs	Chromosome	No. of SNPs
1	694 366	11	258 748
2	520 483	12	395 053
3	507 286	13	397 581
4	476 118	14	345 482
5	494 216	15	337 079
6	508 735	16	304 953
7	405 410	17	265 557
8	444 234	18	289 416
9	361 325	19	221 786
10	398 909	X	222 912

### Mathematical modelling of radiation-induced chromosomal aberrations in time

The yield-time curve of radiation-induced chromosomal aberrations, indicative of radiosensitivity of late stages of the cell cycle, was modelled by an exponential function in time (Eq. 1), with two parameters: k (gain, responsible for the initial induction of chromosomal aberrations) and T (time constant, related to the speed of the modelled process).

(1) $$G2\left(t\right)=\left\{\begin{array}{c} k*e^{-\frac{t}{T}}\;t\geq 0\\ 0\;\;\;\;t<0 \end{array}\right)$$

The Levenberg-Marquardt (LM) nonlinear least squares method was used for parameter estimation. In the case of a few biological replicates the final parameter value for the mouse strain under investigation was calculated as the average of the values obtained for every biological replicate. The convergence of the LM algorithm was checked to ensure the stability of the parameter estimates. The mean relative absolute residuum was used to assess the quality of model fitting.

### Unsupervised clustering of kinetics parameters - the definition of subgroups

The distributions of both, k and T parameters, and the area under the yield-time curve (AUF) were subjected to Gaussian Mixture Model (GMM) decomposition [19], where the probability density function is presented as a convex combination of the constituent probability functions. GMM allows for the construction of mice subpopulations characterized by different kinetics of the chromosomal aberration yield-time curve and it has the ability to create soft boundaries between clusters accompanied by the probability of belonging to a particular class. The Expectation-Maximization algorithm (EM) was used fo ther maximization of the likelihood function, accompanied by the Bayesian Information Criterion (BIC) for model selection [20-22].

### Selection of candidate relevant SNPs

The standard GWAS projects require the statistical tests to be performed for every SNP independently, and the correction for multiple testing to be applied afterwards to control the level of FWER. Having only a collection of 14 mice, split into two groups, we propose the most conservative approach to be applied. The SNP is going to be classified as a candidate relevant SNP if and only if the allele at that SNP is identical in all the mouse strains assigned to one group, and identical but different from the previous group in all the mouse strains assigned to the other group.

### Along-chromosome distribution of candidate relevant SNPs

To verify the hypothesis of the non-random distribution of candidate relevant SNPs along chromosomes, the r-scan test was applied. The null hypothesis states that the locations of identified candidate relevant SNPs are independent and uniformly distributed along the chromosome. The alternative hypotheses of interest are, first, that the points tend to occur in a clumped fashion, or second, that they tend to occur in a regularly spaced fashion [23,24].

### Identification of nonsynonymous SNPs among candidate relevant SNPs

The selection process led to the identification of candidate relevant SNPs, the most interesting of those being non-synonymous SNPs (nsSNPs). Polymorphisms of this type lead to a change of the amino acid in the protein sequence. To assess the impact of nsSNPs on the organism, widely available programs were used: teh PHANTER *(Protein Analysis Through Evolutionary Relationships) *Classification System [25], PhD-SNP *(Predictor of human Deleterious Single Nucleotide Polymorphisms) *[26], SNAP *(Synonymous Non-synonymous Analysis Program) *[27], SIFT *(Sorting Intolerant From Tolerant) *[28] and PolyPhen *(POLYmorphism PHENotyping) *ver. 2 [29]. Each of them predict, with some probability, whether the amino acid change could cause a deleterious effect. Most of the algorithms use the information about protein evolutionary sequence conservation. Some of them (e.g. PolyPhen) utilize additional information, such as the annotation and protein structure. Additionally, when nsSNPs result in the substitution of amino acids involved in phosphorylation (changing Serine, Threonine or Tyrosine), it is possible to assess the group of protein kinases (PK) that could be blocked in the investigated position. To address this issue, GSP ver. 2.1 *(Group-based Prediction System) *[30] was used.

### *In silico *prediction of radiosensitive mouse strains

The CGD database contains information on genome-wide polymorphisms for 74 mouse strains, fourteen of those were used in our study to construct a set of candidate radiosensitivity relevant SNPs. The remaining 60 mouse strains were subjected to in silico radiosensitivity prediction. The proposed similarity score shows the similarity between the mouse strain under investigation and the pattern defined by the radiosensitive group of mice at candidate relevant SNPs. It ranges from 0 [none of the alleles are identical to the allele in the pattern defined for radiosensitive mice at candidate relevant SNPs) to 100 (alleles for all candidate relevant SNPs ale identical to those in radiosensitive pattern) and is calculated as the percentage of polymorphisms with allele identical to the form observed at candidate relevant SNPs for all radiosensitive mice. The distribution of the similarity score was analysed and the method for outlier detection by Hubert and Vandervieren [31] was applied to detect additional candidate radiosensitive mouse strains. In order to illustrate the relationship between 74 mouse strains, a phylogenetic tree was created using the UPGMA algorithm *(Unweighted Pair Group Method with Arithmetic mean) *[32]. The distance matrix was calculated with the Jukes-Cantor method [33] with evolutionary distance estimated for candidate relevant SNPs only.

## Results

### Mathematical modelling of radiation-induced chromosomal aberrations in time

**Figure 1:**
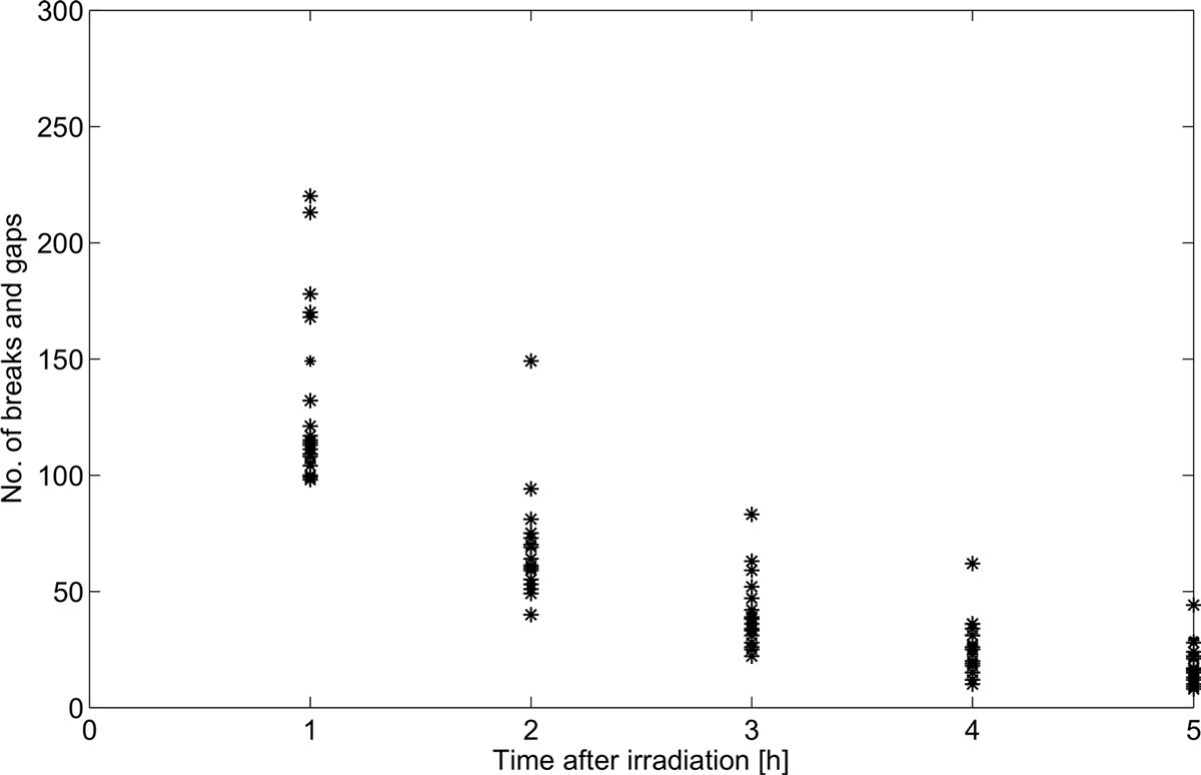
**Graphical presentation of G2CR levels in time for all analysed mouse strains**.

**Figure 2:**
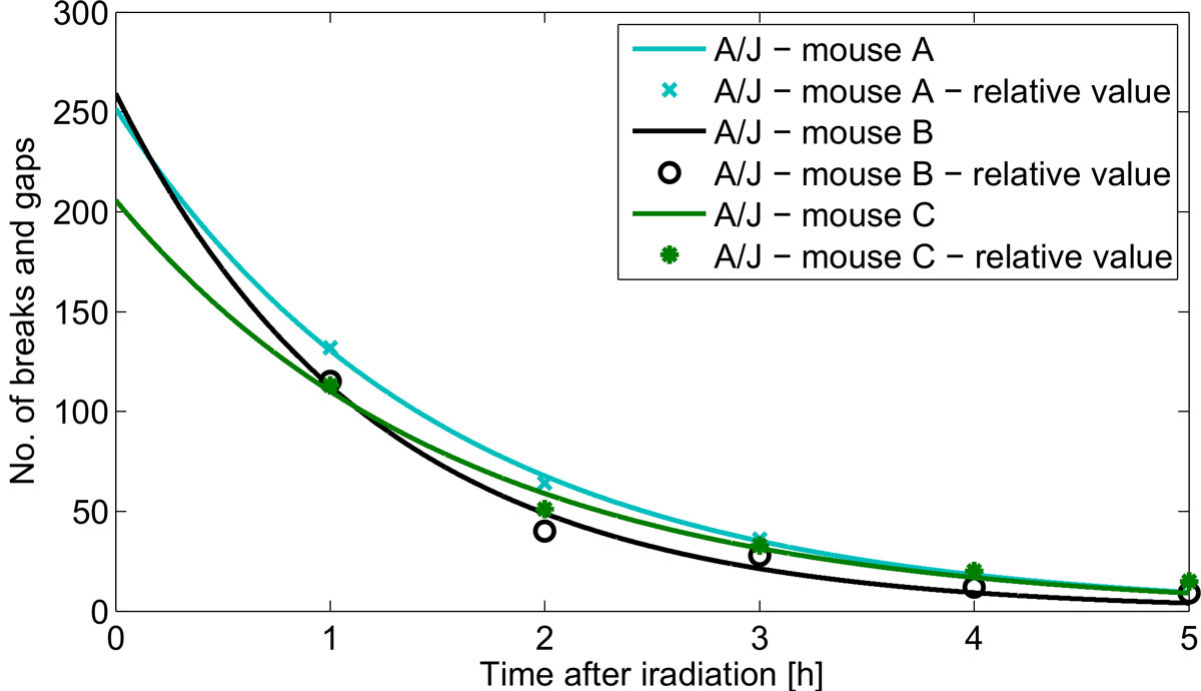
**The exemplary results of model fitting versus raw measurements**. Solid lines present the results of model fitting, while symbols (stars, circle and cross) mark corresponding raw measurements. Each mouse strain is described by different color.

**Figure 3:**
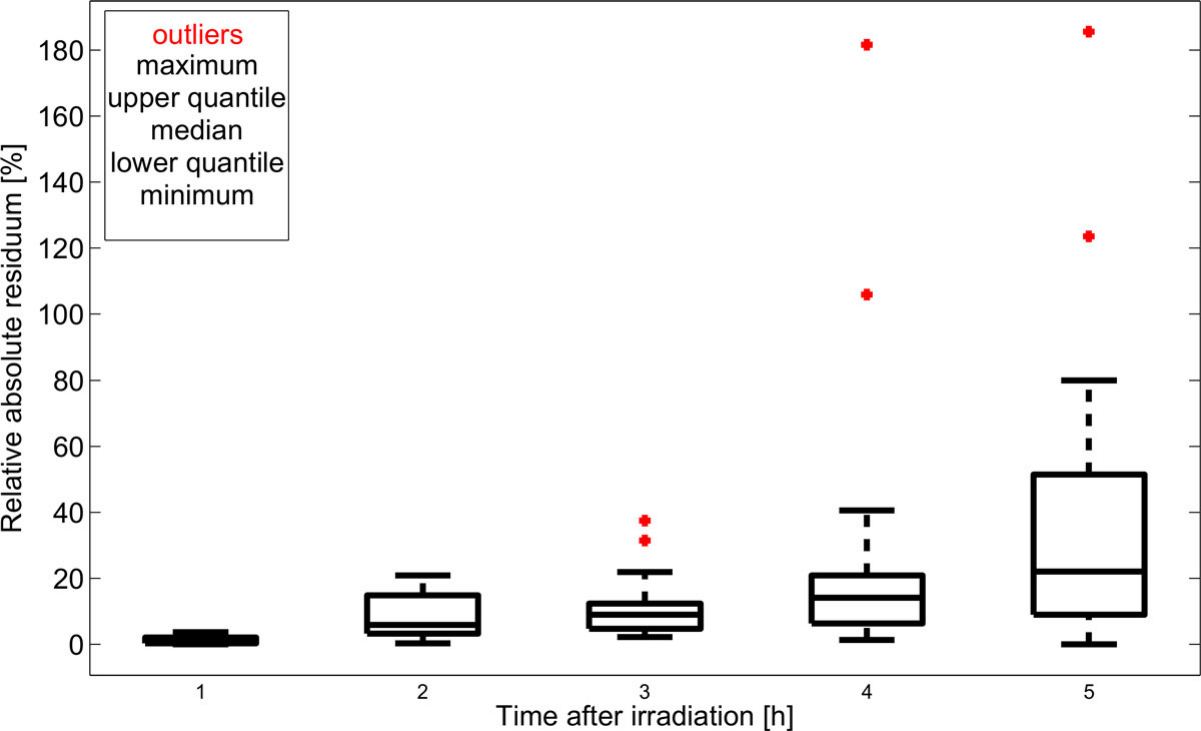
**Boxplots of absolute relative residua for model fitting depending on the time after irradiation**. Horizontal bold line marks median value, upper and lower rectangle sides show upper and lower quartiles respectively, red dots are used to mark the outliers, and whiskers present minimum and maximum values (with outliers being skipped).

The collected G2CR measurements are presented Figure 1. There was no case where the LM nonlinear least squares algorithm was divergent. An example of fitting model for A/J mice is shown in Figure 2. Table 3 gives the estimates of both the gain (k) and time constant (T) parameters for all of the analysed mouse strains. The area-under-function (AUF) values are also included in the table. The absolute relative residua ranged from 1.e3-14% to 185.57%, and were the smallest by average for 1h after irradiation, and the highest for 5h after irradiation. Figure 3 presents their distribution depending on time after irradiation. The observed high error values obtained for 4h and 5h stem from limitations of the assumed mathematical model. In some cases, one can observe the unrepaired breaks and gaps resulting in a non zero steady state value. Since the model curve tends to 0 with time tending to infinity, the relative error increases significantly.

**Table 3:** Estimates of k and T parameters obtained by LM NLS algorithm.

Mouse Strain	k	T	AUF
A/J	239.0	1.44	330.2
AKR/J	190.8	1.60	291.4
**Balb/cAn**	**340.1**	2.31	**696.3**
**Balb/cByJ**	**287.4**	1.85	**497.1**
C3H/HeHsd	188.1	1.81	318.4
CBA/Ca	177.5	1.92	315.3
CBA/H	139.8	3.10	346.8
C57Bl/6J	163.9	2.51	355.0
DBA/2J	154.7	2.18	302.9
LP	193.5	1.87	332.8
NOD/LtJ	220.7	1.47	313.8
**NON/LtJ**	**427.5**	1.14	**508.2**
NZB/B1NJ	202.4	1.44	282.8
SJL/J	178.4	1.90	314.0

### Unsupervised clustering of kinetics parameters - the definition of subgroups

**Figure 4:**
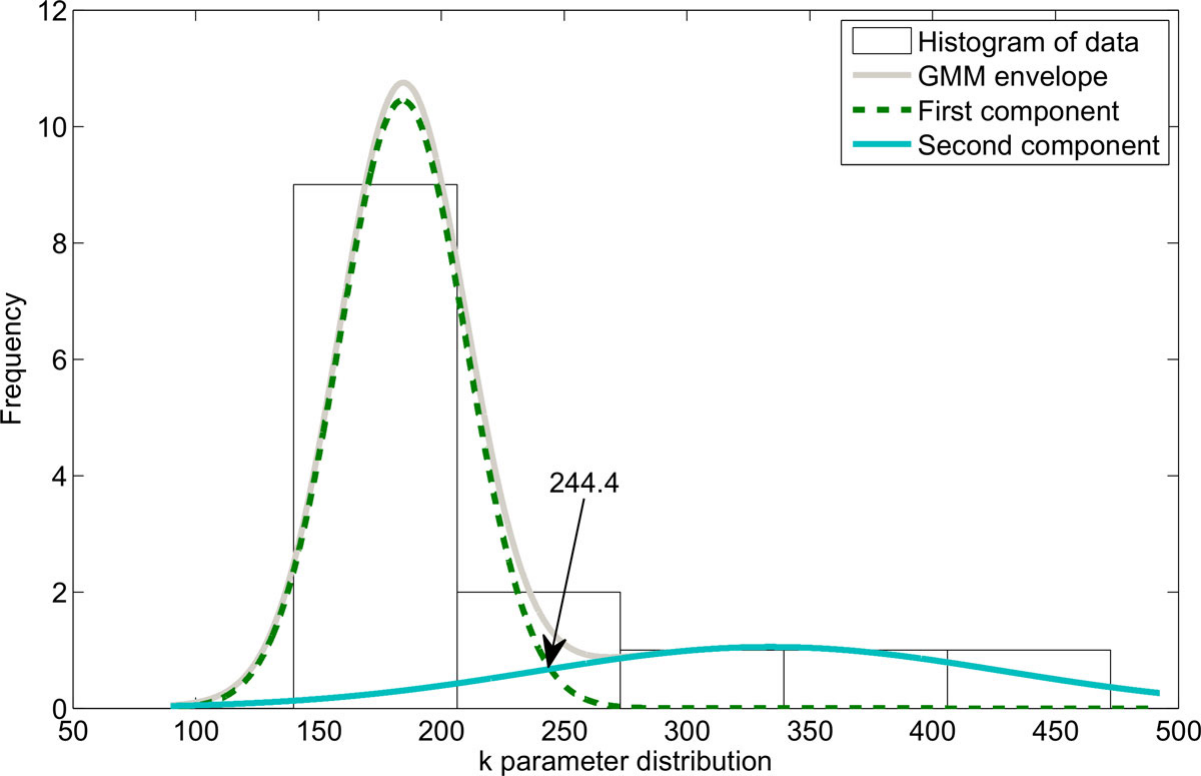
**GMM decompositions of gain k parameter distribution together with obtained threshold value for mouse subpopulation definition**. Green, dashed line and blue solid represent GMM model components, while grey solid line represents GMM envelope.

**Figure 5:**
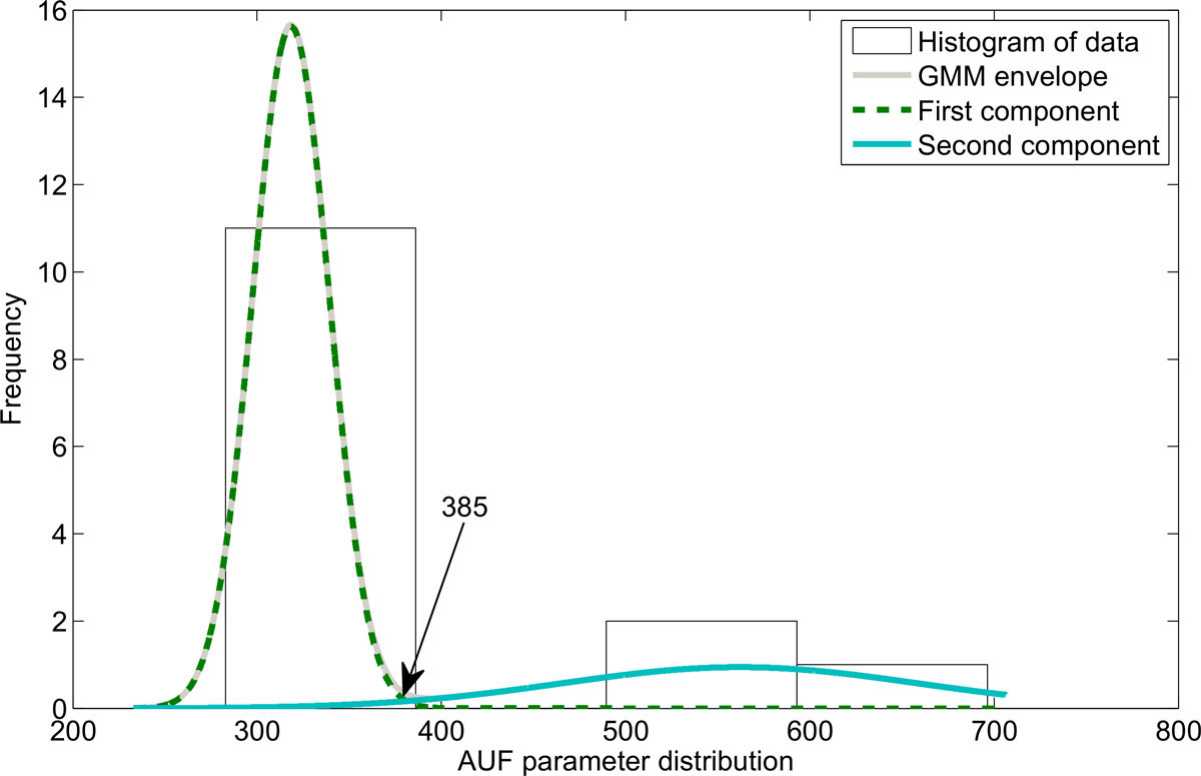
**GMM decompositions of AUF parameter distribution together with obtained threshold value for mouse subpopulation definition**. Green, dashed line and blue solid represent GMM model components, while grey solid line represents GMM envelope.

**Figure 6:**
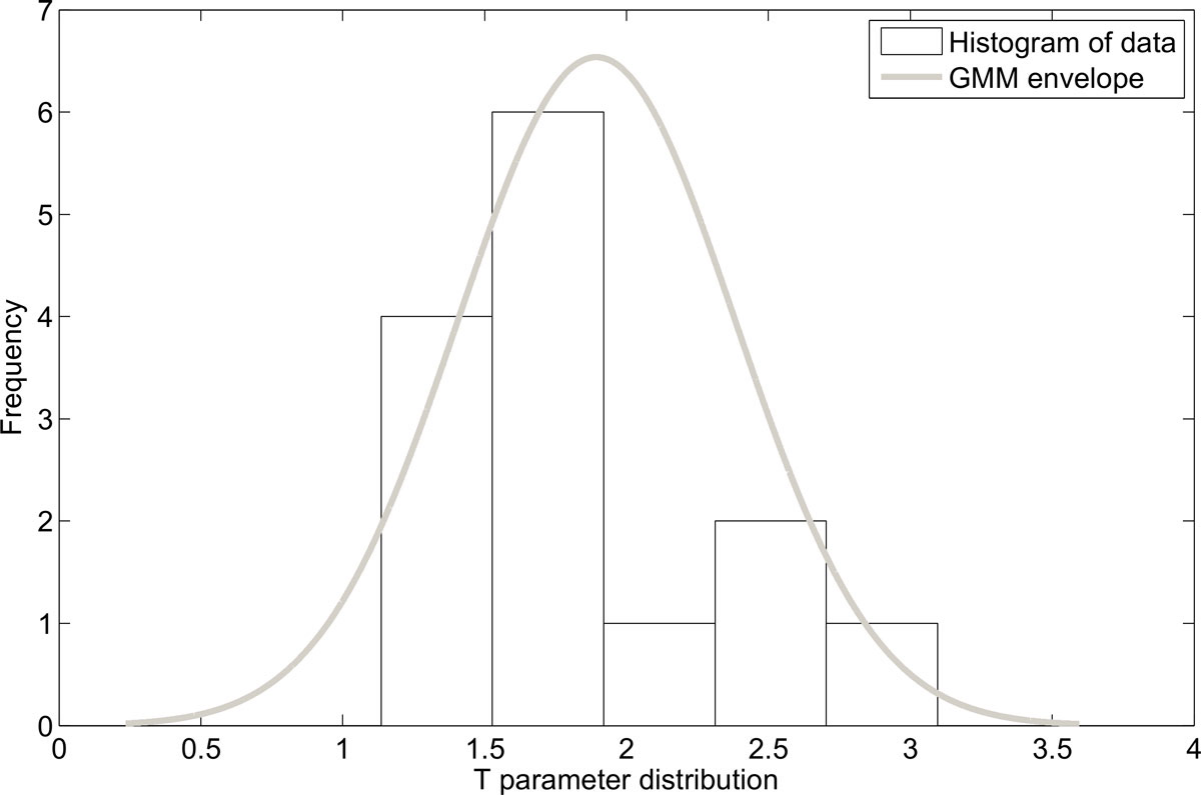
**GMM decompositions of time constant T parameter distribution**. Grey solid line represents GMM envelope.

**Figure 7:**
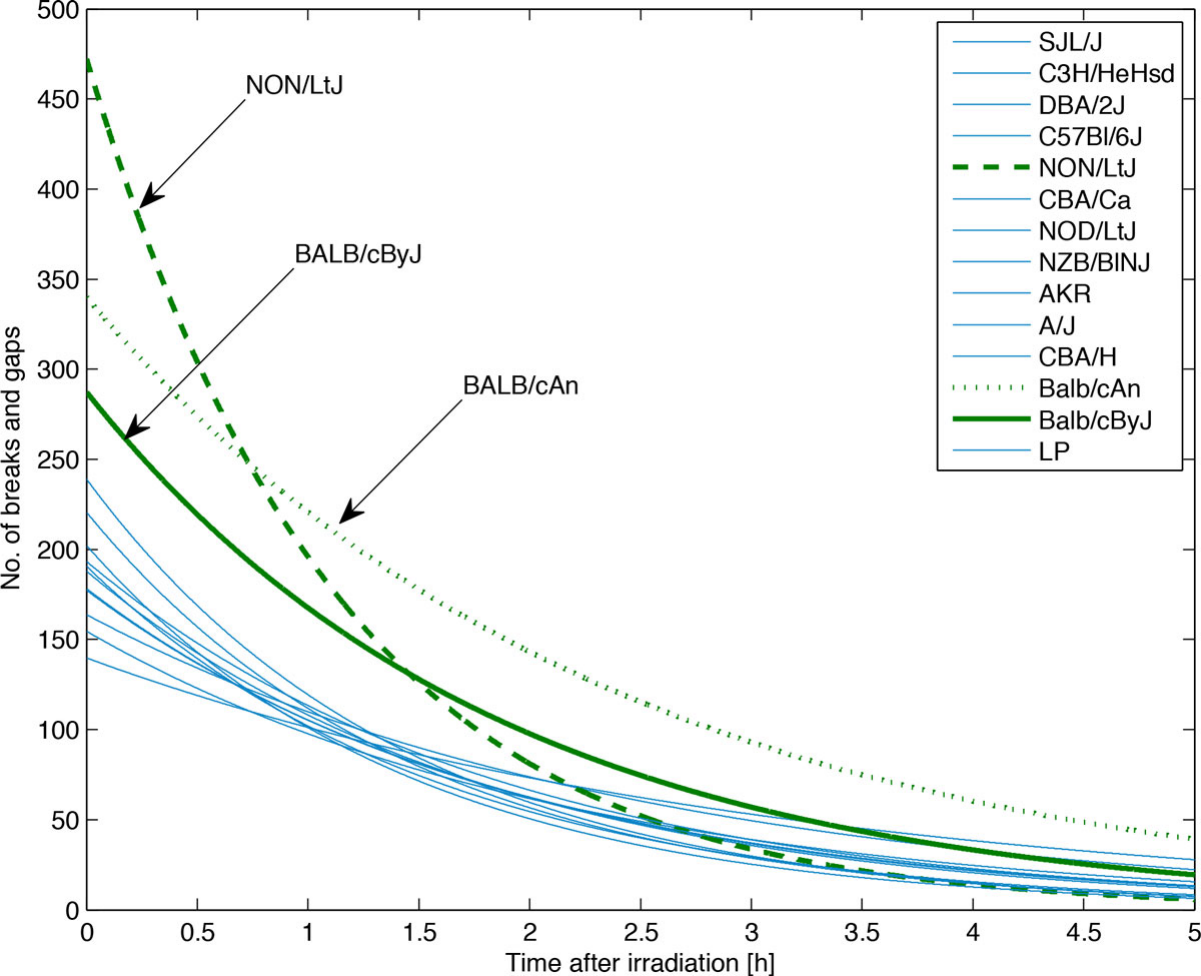
**The final models of the G2CR yield-time curve**. Radiosensitive mouse strains are marked green, while regular response mouse strains are marked blue solid line.

GMM technique was applied to obtain the criteria for mouse classification. Figures 4, 5, and 6 present the histograms of the decomposed parameters together with their mixture model. Maximum conditional probability criterion was used to decide on subpopulation definition, the threshold values obtained were equal to 244.4 for gain (k), and 385 for AUF. The threshold value in the T-domain cannot be identified due to the single component model. The radiosensitive subpopulation of mice was defined as those with a k-value above its threshold or an AUF above its threshold. The second group, named the regular responder subpopulation, consisted of the remaining mouse strains. Figure 7 shows the model yield-time curves of chromosomal aberrations with GMM distinguished mouse subpopulations. The following mouse strains were classified into the radiosensitive group: BALB/cAn, BALB/cByJ and NON/LtJ.

### Selection of candidate relevant SNPs

The SNP selection was performed following the algorithm described in the Materials and Methods section, and 1857 candidate relevant SNPs were found. Because of different reference/variant frequencies at every SNP, it is not possible to provide the exact number of the candidate relevant SNPs expected by chance. Assuming that the variant allele is observed among radiosensitive mice, and the reference allele is present in regular response mice, the probability of such a configuration is equal to *p*^3 ^(1 − *p*)^11^, where p stands for the variant allele population frequency. The overall variant frequency, obtained as a weighted average of variant frequencies at every chromosome (Table 4), is equal to 9.62%. So the expected number of relevant SNPs found by chance is equal to (0.0962)^3^(1 − 0.0962)^11 ^7,849,649 = 2297 and is higher than the number found as candidate relevant SNPs. However, a detailed inspection performed for every chromosome independently shows that there are some chromosomes with overrepresentation of found candidate relevant SNPs, while there were no candidate SNPs found for some of the other chromosomes (Table 5).

**Table 4:** Estimates of variant allele frequency, pSNPs stands for polymorphic loci.

Chr.	No. of SNPs	No. of pSNPs	pSNPs variant freq [%]	Overal Variant Freq [%]	Chr.	No. of SNPs	No. of pSNPs	pSNPs variant freq [%]	Overal Variant Freq [%]
1	694366	341063	22.62	11.11	11	258748	146431	21.39	12.11
2	520483	262264	19.99	10.07	12	395053	198106	20.62	10.34
3	507286	229264	23.42	10.59	13	397581	181873	20.01	9.16
4	476118	219418	21.97	10.13	14	345482	213003	20.98	12.94
5	494216	211104	21.05	8.99	15	337079	150984	20.91	9.37
6	508735	232322	20.74	9.47	16	304953	114988	19.99	7.54
7	405410	207630	19.50	9.99	17	265557	135794	21.48	10.98
8	444234	218490	18.36	9.03	18	289416	119201	21.14	8.71
9	361325	183990	17.64	8.89	19	221786	94203	20.53	8.72
10	398909	124856	20.10	6.34	X	222912	44286	24.91	4.95

**Table 5:** Distribution of relevant SNPs along the chromosomes.

Chr.	No. of SNPs	Variant Freq [%]	No. of expected by chance relevant SNPs	No. of found candidate relevant SNPs	Fisher test p-value	Chr.	No. of SNPs	Variant Freq [%]	No. of expected by chance relevant SNPs	No. of found candidate relevant SNPs	Fisher test p-value
1	694366	11.11	260	322	0.0017	11	258748	12.11	111	19	*<*1e-6
2	520483	10.07	165	0	*<*1e-6	12	395053	10.34	131	113	ns
3	507286	10.59	175	6	*<*1e-6	13	397581	9.16	106	89	ns
4	476118	10.13	152	0	*<*1e-6	14	345482	12.94	163	0	*<*1e-6
5	494216	8.99	127	106	ns	15	337079	9.37	93	65	0.0157
6	508735	9.47	144	12	*<*1e-6	16	304953	7.54	55	2	*<*1e-6
7	405410	9.99	126	74	0.0001	17	265557	10.98	98	16	*<*1e-6
8	444234	9.03	115	281	*<*1e-6	18	289416	8.71	70	0	*<*1e-6
9	361325	8.98	92	0	*<*1e-6	19	221786	8.72	54	17	6.3e-6
10	398909	6.34	49	724	*<*1e-6	X	222912	4.95	15	0	3.1e-5

### Along-chromosome distribution of candidate relevant SNPs

**Figure 8:**
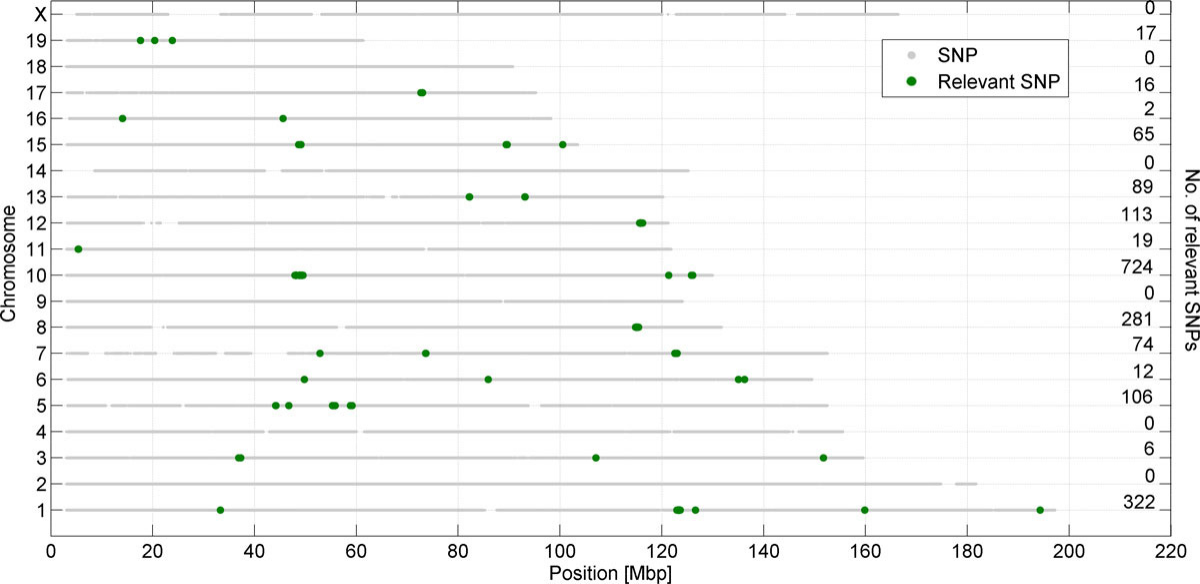
**The candidate relevant SNP clusters along chromosomes**. Plot presents the location of detected clusters of relevant SNPS (marked as green dots) at particular chromosome. Number of relevant SNPs located across the chromosome is showed on the right side of panel.

As seen in Table 5, there are some chromosomes with a significantly higher number of candidate relevant SNPs and other chromosomes with a significantly lower number of that type of loci. This suggests that there are chromosomes with a clumped distribution of relevant polymorphic loci. R-scan test was applied to verify the hypothesis of the uniformity of location. Applying the r-scan test (with r equal to 1) to the distribution of candidate relevant SNP locations along chromosomes gives p-values less than 1e-12 for every chromosome, and allows for the rejection of the null hypotheses. Figure 8 shows a graphical illustration of the distribution of candidate relevant SNPs' locations. Candidate relevant SNPs concentrate in 29 clusters located in 28 genes. The list of these genes together with a functional classification of candidate relevant SNPs is presented in Table 6. Among 1857 candidate relevant SNPs, 48 are located in exons, 880 in introns, 14 in UTR regions, and 915 in intergenic regions. Two genes, Htr5b and Ccdc93 seem to be at highest risk of their product being modified.

**Table 6:** The gene location of candidate relevant SNPs and their functional classes.

			Functional-class				
			
Gene symbol	Gene ID	No. of candidate relevant SNPs	Intron variant	missense	synonymous-codon	upstream variant 2kb	UTR variant 3/5 prime
Grik2	14806	422	422	-	-	-	-
Cntnap4	170571	144	143	-	-	1	-
Msh3	17686	80	76	2	2	-	-
Ccdc93	70829	72	55	1	2	6	8
Sox6	20679	40	40	-	-	-	-
Cc2d2a	231214	37	32	1	4	-	-
Htr5b	15564	34	9	3	9	10	3
Galnt6	207839	15	13	1	-	-	1
Apba1	319924	15	15	-	-	-	-
Syt10	54526	14	14	-	-	-	-
Alk	11682	10	10	-	-	-	-
Fbx15	242960	9	8	-	-	1	-
Insig2	72999	6	3	-	-	3	-
Srgap1	117600	5	5	-	-	-	-
Gip2	54120	3	3	-	-	-	-
Bst1	12182	2	1	-	-	1	-
Fgf2	14173	2	1	-	-	-	-
Gprc5a	232431	2	2	-	-	-	-
Npy	109648	2	-	-	-	1	1
2900011O08Rik	67254	1	1	-	-	-	-
4932438A13Rik	229227	1	1	-	-	-	-
Hsd17b14	66065	1	1	-	-	-	-
Kcnh1	16510	1	1	-	-	-	-
Pcsk5	18552	1	1	-	-	-	-
Phactr2	215789	1	1	-	-	-	-
Rab12	68708	1	-	-	-	-	1
Slc9c1	208169	1	1	-	-	-	-
Znrf3	407821	1	1	-	-	-	-

### Identification of nonsynonymous SNPs among candidate relevant SNPs

Eight SNPs, located in 5 genes, appeared to be nonsynonymous (nsSNP) alerting the amino acid sequence of a protein. Detailed information is presented in Table 7 while Table 8 gives more data on the predicted impact of detected nonsynonymous SNPs on protein function. Nonsynonymous SNPs resulting in the substitution of amino acids involved in the process of phosphorylation, were checked with the GPS algorithm in order to predict their effect on protein kinases (PK). Two nsSNPs, rs48840878 (Msh3) and rs5144199 (Cc2d2a) were predicted as having increased probability of a deleterious effect, with one of them capable of disordering phosphorylation with 14 PKs (Table 9). All of above mentioned genes had their gene ontology terms and signalling pathway participation analyzed. Msh3 has 28 major GO terms assigned, among which the most relevant in a radiosensitivity context are DNA repair, mismatch repair, or meiotic mismatch repair (GO:0006281, GO:0006298, GO:0000710). The KEGG database brings up two signal pathways related to cancer development in humans (hsa05200, hsa05210) with Msh3 involved. There are reports in the literature on other Msh3 polymorphisms involved in radiosensitivity of breast cancer patients [34]. Htrb5 has 12 GO terms specified, mainly related to G-protein coupled receptor activity and signaling pathway (GO:0004930, GO:0007186) and serotonin binding, receptor activity, and signaling pathway (GO:0051378, GO:0004993). G-protein-coupled receptors (GPCRs) have recently emerged as crucial players in tumour growth and metastasis [35], there are many reports on the active role of serotonin in cancer development [36,37]. Cc2d2a gene is related to cancerogenesis through involvement in the Smoothened signalling pathway (GO: GO:0007224) [38]. Mutations in gene Ccdc93 are associated with several cancers in humans, with similar observations are for Galnt6, which is probably involved in breast cancerogenesis through O-glycan processing (GO:0016266) [39].

**Table 7:** Nonsynonymous SNPs found among candidate relevant SNPs.

nsSNP	Chr.	Position	Gene	Gene ID	NT Change	AA Change	Protein position
rs30775581	1	123424576	Htr5b	15564	A→G	Val→Ala	64
rs30775583	1	123424459	Htr5b	15564	G→A	Ala→Val	103
rs30776464	1	123424420	Htr5b	1556	A→G	Val→Ala	116
rs30776698	1	123379867	Ccdc93	70829	A→G	Ile→Val	432
rs51441999	5	44121147	Cc2d2a	231214	G→A	Ala→Thr	1288
rs51455675	13	93079452	Msh3	17686	C→T	Val→Ile	464
rs48840878	13	93079409	Msh3	17686	G→A	Ser→Phe	478
rs31954017	15	100527632	Glant6	207839	G→A	Arg→Gln	473

**Table 8:** Predicted impact of detected nonsynonymous SNPs on protein function.

nsSNP	Gene	PANTHER subSPEC	SNAP RI	SIFT Score	SIFT MSC	SIFT NSatP	PolyPhen Score	PolyPhen Sensitivity	PolyPhen Specificity	PhD SNP RI
rs30775581	Htr5b	**-3.716**	0	0.54	3.56	21	0.001	0.99	0.15	2
rs30775583	Htr5b	**-4.093**	4	0.33	2.99	28	0.130	0.93	0.86	6
rs30776464	Htr5b	no data available	1	0.59	3.17	13	0.000	1.00	0.00	6
rs30776698	Ccdc93	-1.127	5	1.00	4.32	3	0.000	1.00	0.00	7
rs51441999	Cc2d2a	-2.701	5	0.48	3.08	24	0.006	0.97	0.75	8
rs51455675	Msh3	**-3.086**	4	0.77	3.03	25	0.002	0.99	0.30	4
rs48840878	Msh3	**-3.452**	2	0.31	3.03	25	**0.726**	**0.86**	**0.92**	2
rs31954017	Glant6	**-4.150**	6	1.00	3.03	56	0.000	1.00	0.00	6

subSPEC - substitution position-SPecific Evolutionary Conservation; RI - Reliability Index, MSC - Median of Conservation Value, NSatP - Number of Sequences at Position

**Table 9:** Protein kinases predicted to be effected by Msh3 phosphorylation related to nsSNP rs48840878 and FNLEKMLSKPESFKQ protein sequence.

Kinases	Score	Cutoff
CAMK	1.566	1.430
CAMK/PHK	2.476	2.048
STE/STE20	1.779	1.632
AGC/PKG/PKG1	3.938	2.875
AGC/RSK	1.211	0.895
CAMK/CAMK1/CAMK4	5.100	4.900
CAMK/CAMK2/CAMK2a	2.417	1.806
CMGC/GSK/GSK3A	2.833	2.667
STE/STE7/MAP2K1	6.000	5.000
Other/CK2/CK2b	5.000	5.000
Other/NEK/NEK2	7.667	4.333
AGC/PKC/Eta/PKCh	3.750	3.750
CMGC/MAPK/p38/MAPK13	6.667	5.667
STE/STE20/PAKA/PAK3	1.800	1.400

### *In silico *prediction of radiosensitive mouse strains.

**Figure 9:**
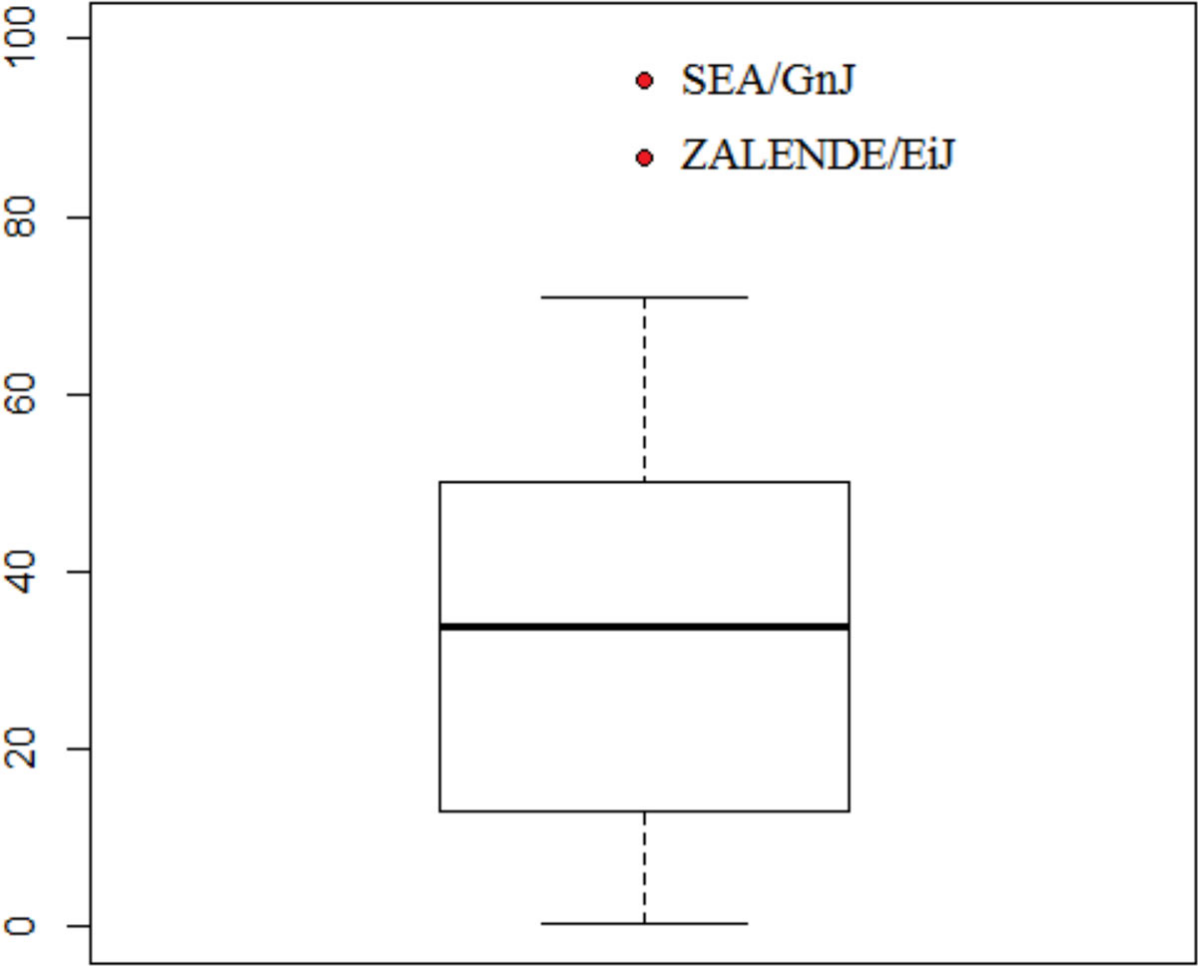
**The boxplot of candidate relevant SNP similarity score, constructed for remaining 60 CGD mouse strains**. Horizontal bold line marks median value, upper and lower rectangle sides show upper and lower quartiles respectively, red dots are used to mark the outliers, and whiskers present minimum and maximum values (with outliers being skipped).

**Figure 10:**
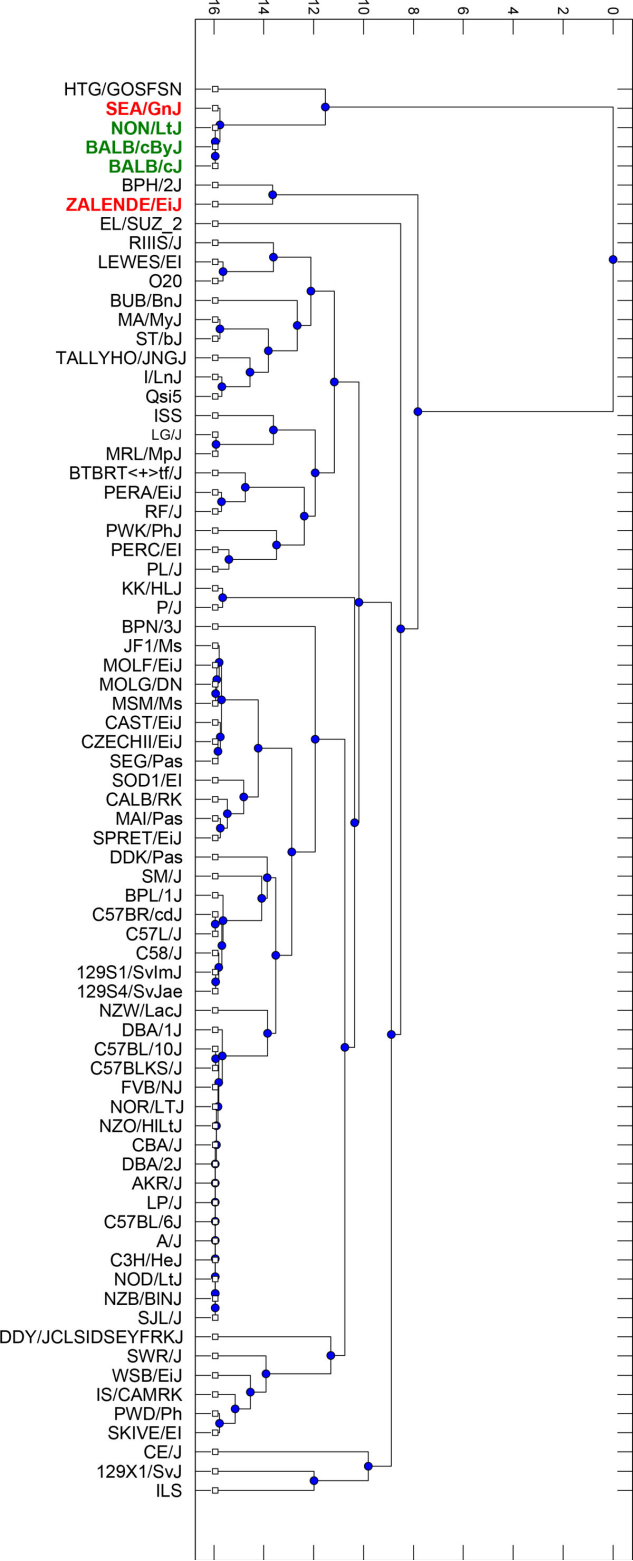
**Candidate relevant SNPs based phylogenetic tree of CGD mouse strains**. GMM distinguished radiosensitive mouse strains are marked green, in silico predicted radiosensitive mouse strains are marked red, remaining CGD mouse strains are marked black.

In silico analysis of candidate relevant SNP similarity score distribution among the remaining 60 CGD mouse strains allowed for the identification of two mouse strains with genetic profiles very similar to the profile of GMM distinguished radiosensitive mice (BALB/cAn, BALB/cByJ and NON/LtJ) - Figure 9. The most similar was SEA/GnJ which has a 95.26% genetic consistency with the radiosensitive pattern. The second in line, was ZALENDE/EiJ, with a similarity of 86.53%. The phylogenetic tree, constructed in the domain of candidate relevant SNPs, shows an evolutionary relationship among analysed CGD mouse strains (Figure 10).

## Summary and conclusions

The proposed strategy for data analysis, which is a combination of mathematical modelling and data mining techniques, allows for the discovery of candidate, trait relevant SNPs in the case of small sample sizes. The effectiveness of the designed methodology was demonstrated for the exemplary problem of seeking the genetic background of radiosensitivity. From the group of candidate relevant SNPs and genes related to those, the analysis revealed that two genes are, according to the literature study, highly significant for the analyzed phenomena of radiosensitivity. One might be responsible for the process of DNA damage repair. The second is indirectly responsible for cell adhesion and was observed to be up-regulated in breast cancer patients. To increase the power of the performed analyses, the biological validation of the obtained putatively relevant SNPs is necessary.

## Competing interests

The authors declare that they have no competing interests.

## Authors' contributions

All authors have made equal contributions to the conception and design of the study, drafting the article or revising it critically for important intellectual content and final approval of the version to be submitted.

## Declarations

Publication of this article has been funded by NCN grant Harmonia 4 no. UMO-2013/08/M/ST6/00924.

This article has been published as part of *Theoretical Biology and Medical Modelling *Volume 11 Supplement 1, 2014: Selected articles from the 1st International Work-Conference on Bioinformatics and Biomedical Engineering-IWBBIO 2013. The full contents of the supplement are available online at http://www.tbiomed.com/supplements/11/S1.
